# Emerging Stroke Risk Factors: A Focus on Infectious and Environmental Determinants

**DOI:** 10.3390/jcdd11010019

**Published:** 2024-01-11

**Authors:** Sajid Hameed, Nurose Karim, Mohammad Wasay, Narayanaswamy Venketasubramanian

**Affiliations:** 1 Department of Neurology, University of Virginia, Charlottesville, VA 22903, USA; drsajidhameed92@gmail.com; 2 Department of Neurology, East Carolina University, Greenville, NC 27834, USA; nurosekarim@live.com; 3 Department of Neurology, Aga Khan University, Karachi 74800, Pakistan; mohammad.wasay@aku.edu; 4 Raffles Hospital, Singapore 188770, Singapore

**Keywords:** stroke, risk factors, air pollution, gut microbiota, high altitude, systemic infection

## Abstract

This review focuses on emerging risk factors for stroke, including air pollution and climate change, gut microbiota, high altitude, and systemic infection. Up to 14% of all stroke-associated mortality is attributed to air pollution and is more pronounced in developing countries. Fine particulate matter and other air pollutants contribute to an increased stroke risk, and this risk appears to increase with higher levels and duration of exposure. Short term air pollution exposure has also been reported to increase the stroke risk. The gut microbiota is a complex ecosystem of bacteria and other microorganisms that reside in the digestive system and affect multiple body systems. Disruptions in the gut microbiota may contribute to stroke development, possibly by promoting inflammation and atherosclerosis. High altitudes have been associated with erythrocytosis and cerebrovascular sinus thrombosis, but several studies have reported an increased risk of thrombosis and ischemic stroke at high altitudes, typically above 3000 m. Systemic infection, particularly infections caused by viruses and bacteria, can also increase the risk of stroke. The risk seems to be greatest in the days to weeks following the infection, and the pathophysiology is complex. All these emerging risk factors are modifiable, and interventions to address them could potentially reduce stroke incidence.

## 1. Introduction

**Figure 1:**
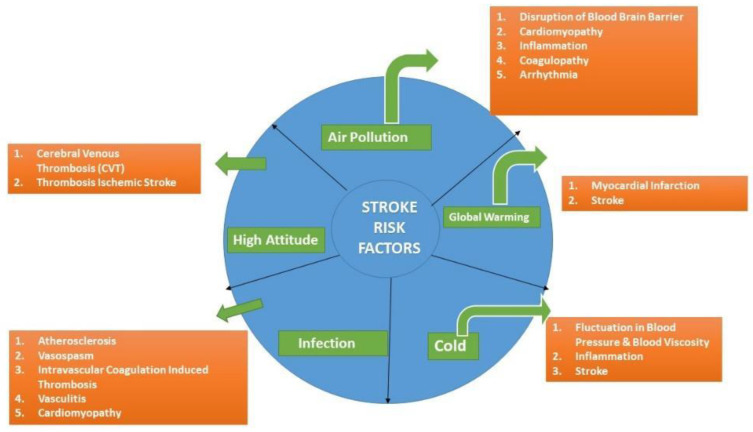
A graphic representation of emerging non-traditional risk factors for stroke.

Stroke burden is a significant global health concern, ranking as the second leading cause of mortality and the third leading cause of disability globally [1]. Unfortunately, this stroke burden is escalating at an alarming rate. Over the last three decades, there has been a 70% increase in stroke incidence, an 85% increase in prevalence, a 43% increase in mortality, and a 32% increase in disability-adjusted life years (DALYs) [1,2]. This is largely attributed to shifts in stroke trends in low- and middle-income countries (LMICs). In 2019, the age-standardized stroke-related mortality rate was 3.6 (3.5–3.8) times greater in the World Bank’s low-income group compared to its high-income group [2]. People under the age of 70 years diagnosed with a stroke represent another subgroup, which is experiencing an increase in both incidence (22% increase) and prevalence (15% increase) over the same period. This suggests that risk factors beyond old age are important that should be identified and addressed [1,2,3]. Besides the traditional vascular risk factors, newly identified environmental and infectious risk factors are gaining importance in recent years. This narrative reviews four non-traditional emerging risk factors for stroke: air pollution and climate change, gut microbiota, high altitude, and systemic infections (Figure 1):

## 2. Methodology

We conducted a comprehensive literature search across multiple databases (MEDLINE/Pubmed, Embase, Web of Science Core Collection, and Google Scholar) to identify pertinent articles published in the last five years that discuss non-traditional stroke risk factors. The review process was iterative, with insights from initial articles informing the search and selection of subsequent articles. This approach ensured a thorough and rigorous review of the current literature on non-traditional stroke risk factors. Ultimately, we shortlisted and focused on four emerging risk factors for stroke: air pollution and climate change, gut microbiota, high altitude, and systemic infections. The selection of these risk factors was subjective and based on their relevance to stroke.

## 3. Air Pollution

Air pollution is a known risk factor for cardiovascular disease. However, its association with the cerebrovascular disease including both ischemic and hemorrhagic stroke is under-studied and under-reported. Air pollution is an emerging risk factor and reportedly accounts for 14% of all stroke-associated mortality [4]. Its incidence is more pronounced in LMIC [5]. Air pollution is broadly classified into household air pollution and ambient or outdoor air pollution, which has been primarily studied in association with stroke [6]. It consists of small particles and several toxic gases. Particles based on their size, particulate matter (PM) are classified as coarse (<10 μm), fine (<2.5 μm), and ultrafine (<1 nm). Larger particles are mostly industrial emission whereas smaller particles include traffic pollution. Common toxic gases include carbon monoxide, black carbon, nitrous oxide, sulfate, and ozone.

Short term exposure includes daily and hourly variation of air pollution concentration. There have been conflicting country-wise results regarding the association between short term exposure to air pollution and ischemic stroke. In the US, they found a small but statistically significant increase in the risk of ischemic stroke admission of 1.03% per IQR increase (22.96 μg/m^3^) in PM10 concentration 1–2 days before stroke onset [7]. A similar study in China also reported 0.29% increase in hospital admissions for ischemic stroke per 10 μg/m^3^ increased PM2.5 on the day of stroke [8]. On the contrary, studies in Europe and North America did not show similar results. This difference could be due to the geographic risk of exposure to air pollution and the hourly and daily exposure level. Long term exposure includes weeks to years of cumulative exposure to air pollution and its overall effect on population and the incidence of ischemic stroke.

There have been limited studies on the association between air pollution and hemorrhagic strokes (including both intracerebral hemorrhage and subarachnoid hemorrhage). A study carried out in Taiwan showed 12% risk increase in intracerebral hemorrhage admissions per 17.46 μg/m^3^ (IQR) increased PM2.5 on the day of stroke [9]. Another study performed in Algarve showed a 5.7% (95% CI 1.02–1.10; *p* = 0.002) increased risk of spontaneous intracerebral hemorrhage per 10 μg/m^3^ increase in PM2.5 concentration. Further, the risk of non-lobar hemorrhage was higher than lobar hemorrhage [10].

The pathophysiology of air pollutants leading to stroke is complex and multifaceted. In vitro laboratory studies involving cultures of neuron and glial cells have demonstrated an increased cellular vulnerability to oxygen and glucose deprivation, changes in synaptic function, and an increase in inflammatory cytokines when exposed to PM air pollutants [11,12,13]. The potential harm of PM is inversely proportional to its size, as smaller particles can penetrate deeper into the lungs and have larger reactive surfaces. Cardiovascular studies have found a stronger correlation between diseases and PM with a diameter less than 2.5 μm compared to larger PM [14]. PM also adsorbs various organic chemicals and heavy metals. Among these organic chemicals, polycyclic aromatic hydrocarbons (PAHs) and persistent organic pollutants (POPs) are recognized as activators of the aryl hydrocarbon receptor (AHR). AHR, a ligand-activated transcription factor, triggers the translation of various target genes, such as CYP1A1 and CYP1B13. These genes play crucial roles in several cellular functions, including the formation of reactive oxygen species, detoxification, proliferation, and immune regulation. Over time, POPs accumulate in body fat, leading to chronic toxicity [15,16].

Different animal models have been used to investigate the relationship between air pollution and stroke. One study demonstrated that the mice exposed to diesel exhaust exhibited an upregulation of interleukin 6, leading to platelet activation and an increase in tissue factor release, fibrinogen, and factor VIII [17]. Similarly, another study studying effects of vehicle exhaust exposure in apolipoprotein E knockout mice revealed a disruption of their blood–brain barrier [18]. In healthy adults, diesel exposure has been linked to platelet aggregation and thrombus formation at sites of vascular injury [19]. Air pollution has also been associated with endothelial cell apoptosis and a reduction in circulating levels of endothelial progenitor cells [20,21]. One hypothesis suggests that air pollution could influence stroke risk by affecting blood coagulability, thereby increasing an individual’s susceptibility to acute stroke events. The free radicals generated by air pollutants could trigger inflammatory responses and enhance blood coagulation and plasma viscosity, thereby increasing the risk of ischemic stroke but not hemorrhagic stroke. There have been studies showing an association between large artery atherosclerosis as well as an increase in the small vessel atherosclerosis with exposure to various toxins, including nitrogen oxide and sulfur dioxide [21]. Exposure to ozone was associated with cardioembolic stroke [22].

During the first peak of COVID-19, we observed a significant decrease in stroke admission during the lockdown [23,24,25]. This reduction may be due to reluctance to go to the hospital for the fear of contracting infection or the actual stroke incidence may have reduced due to less exposure to environmental air pollutants. We need more prospective studies to confirm this.

Currently, 91% of the population lives in areas where exposure to air pollution is above the threshold set by WHO [6]. Even if the air pollutant exposure is below the levels set by WHO, there is still an association with an increased risk of ischemic and hemorrhagic stroke [5,26]. Therefore, we need to make a conscious effort to work on this modifiable and emerging risk factor for stroke.

## 4. Climate Change

Climate change is defined as changes in weather patterns and is associated with significant adverse health outcomes. The Lancet Countdown on Health and Climate Change describes “climate change as the greatest global health threat facing the world in the 21st century” [27]. Global warming is a direct consequence of elevated greenhouse gas levels. This results in the retention of infrared radiation, thereby warming the atmosphere and the Earth’s surface. An increase in temperature due to global warming has been linked to a 50% rise in heat-related deaths among individuals aged over 65 years [28]. These deaths are linked to myocardial infarctions, strokes, and other mental health and neurological events [29]. On the other hand, cold-related morbidity has been attributed to cardiovascular stress, which is related to changes in blood pressure, blood viscosity, plasma fibrinogen levels, vasoconstriction, and associated inflammatory responses. The 2021 Global Burden of Disease study validated the risks of stroke associated with extreme temperatures [30]. Both extremely high and low temperatures have been linked to an increase in stroke incidence and mortality [31]. Global warming-induced temperature extremes can trigger geomagnetic storms, which have been associated with elevated stroke risk [32]. Furthermore, wildfires have also been linked to cardiovascular mortality and stroke [33,34,35,36].

Several reviews and meta-analyses in the recent literature have discussed the relationship between temperature and stroke risk. One study suggested an acute effect of extreme cold and hot temperatures on stroke risk, with men being more susceptible to extreme heat and women to extreme cold temperatures [37]. This was further substantiated by a systematic review comprising 24 studies that confirmed the increased stroke risk associated with extreme temperatures, both hot and cold [38]. A separate review, comprising 58 studies, reported an overall increase in stroke and stroke-related mortality during periods of extremely hot weather [39]. Similarly, a study from the USA found that an increase in average daily ambient temperature was significantly associated with increased stroke mortality and decreased physical activity [40].

Research conducted in Asia by Zhou et al. discovered a link between exposure to both heat and cold temperatures and the incidence of stroke [41]. In a similar vein, Lei et al. reported an increase in stroke mortality associated with cold spells [42], while Song et al. identified stroke as the second leading cause of loss of disability-adjusted life years (DALYs) due to low temperature [43]. Finally, a study by Kang et al. found a significant correlation between long-term exposure to temperature variability and stroke incidence [44].

The mechanism of stroke due to temperature extremes is not well understood and has been extensively debated. It is suggested that high temperatures may lead to vasodilation, sweating, dehydration, volume depletion and electrolyte imbalances. Other proposed mechanisms include hemoconcentration, thrombocytosis, leukocytosis, and hypercoagulable states. Elevated core body temperature may trigger endothelial inflammation and sympathetic activation, leading to cardiac arrhythmias [45,46]. Among various physiological homeostatic mechanisms, heat shock proteins (HSPs) play a significant role in response to extreme temperatures and have been linked to the risk of ischemic stroke. Kobzeva et al. studied the association between HSPA8 gene variations and the risk of AIS. Two specific SNPs in the HSPA8 gene, rs10892958 and rs1136141, were found to be associated with an increased risk of IS. It is possible that alterations in HSPs induced by extreme temperatures may play a part in increasing the risk of stroke [47].

## 5. Microbiota–Gut–Brain Axis

Gut microbiota is a community of microorganisms residing in our gastrointestinal tract, predominantly in the large intestine. Gut microbiota, comprising trillions in number, includes a diverse array of hundreds of species coexisting in a commensal manner [48,49]. The gastrointestinal environment provides a supportive habitat for the microbiota, which, in turn, synthesizes various useful metabolites and also prevents the entry of other microorganisms into our systemic circulation. Gut microbiota typically establish themselves within the first two years after birth but their composition dynamically evolve with age, dietary habits, antibiotics use, and overall health status [48,49].

The relationship between the gut microbiota, gut–brain axis, and stroke is a growing area of interest in the medical fraternity. The gut–brain axis is a complex system that operates bidirectionally. Vagus nerve (cranial nerve X) is considered to play an important part in this bidirectional communication [50]. The gut microbiome impacts the central nervous system (CNS) and immune system by releasing various metabolites, such as neurotransmitters, neuropeptides, metabolites, and immune factors. These metabolites interact with brain cells either directly crossing the blood–brain barrier or indirectly by acting on the afferent vagus nerve. These interactions are suggested to play a role in improving or worsening neurological disorders [50,51,52]. On the other hand, the CNS also communicates with the gut to control its functions, including intestinal peristalsis, mucus secretion, and the mucosal immune environment. Additionally, the CNS modulates the composition of the gut microbiota through the autonomic nervous system, mainly via the vagus nerve [50,51].

Recent studies have suggested a role for gut microbiota in the development of brain disorders including stroke [48,49,50,51,52]. Antibiotic-induced changes to gut microbiota can impact stroke outcomes. In a mouse model, the antibiotic-treated mice reportedly had a higher mortality in the acute post-stroke period [53]. Furthermore, a healthy gut microbiome is responsible for producing most of the small chain fatty acids (SCFAs), such as acetic acid, propionic acid, and butyric acid, from dietary fiber and complex carbohydrates. SCFAs have multiple physiological functions and have been shown to affect stroke management and outcomes in animal models [51,54]. Supplementation with butyric acid has been shown to reduce infarct volume [55]. Trimethylamine N-oxide (TMAO) is another microbiota produced metabolite that is associated with adverse cardiovascular and stroke outcomes. TMAO promotes thrombosis by accelerating atherosclerosis, increasing platelet activity, and precipitating atrial fibrillation [54,56,57]. Tang et al. investigated the association between plasma TMAO concentration and the incidence of cardiovascular/cerebrovascular events in 4007 subjects who were longitudinally observed for a period of 3 years. They reported a positive correlation between plasma TMAO levels and the risk of thrombosis in a dose-dependent manner, with a reported hazard ratio of 2.54 for highest vs. lowest TMAO quartile (CI, 1.96 to 3.28; *p* < 0.001). This correlation was independent of the traditional risk factors associated with cardiovascular and cerebrovascular diseases [58]. SCFAs, TMAO, and other metabolites also alter blood pressure, which is a known risk factor for stroke [56,59]. The hypertensive patients are also reported to have a decreased richness and diversity in their gut microbiota composition [59]. 

Conversely, brain ischemia has been reported to alter the composition and function of gut microbiota [60]. Dominant symbiotic and beneficial microorganisms in a healthy gut, such as Prevotella, Roseburia, Firmicutes, and Fecalibacterium, are reduced in stroke patients and there is an overgrowth of Bacteroidetes [61,62]. Their presence (or absence) also affects the infarct size, with a larger brain infarct volumes are associated with an increase in Bacteroidetes population [62]. These changes are thought to result from modifications in sympathetic release after a stroke, increased intestinal permeability, neuroinflammation, and the disruption of the blood–brain barrier [60,61]. Another study observed microbial taxa in chronically critically ill (CCI) patients. In their study, Ruminococcus, Roseburia, and Clostridiaceae were positively correlated with improved neurological status. Roseburia, known for producing short-chain fatty acids, was found to be depleted. The study also found that A. muciniphila, known for its anti-inflammatory properties, was abundant in patients with positive neurological status, indicating its role in neurorehabilitation [63].

When discussing the gut microbiota–brain axis, we consider the potential of treating or modulating brain disorders through the use of probiotics, prebiotics, or fecal microbiome transplantation (FMT). Probiotics are living gut microorganisms known to protect tissue by altering homeostasis, reducing oxygen free radical production, inhibiting pro-inflammatory cytokines, promoting anti-inflammatory cytokines, and increasing antioxidant pathways [64,65]. They have shown potential in reducing stroke severity and promoting recovery after brain ischemia [50,66]. Prebiotics commonly comprise non-digestible oligosaccharides, which are degraded by gut microbiota. They promote bacterial growth and diversity, regulate intestinal homeostasis, and increase the production of SCFAs [64].

FMT is the transfer of gut microbiota from a healthy donor to a patient. It has shown potential in relieving symptoms in Parkinson’s disease and autism. Despite the limited literature on FMT’s impact on stroke, it has shown potential in improving stroke outcomes by reducing neurological impairment, brain edema, infarct volume, and intestinal leakage [65]. In a mouse model, a higher Firmicutes to Bacteroidetes ratio, considered neuroprotective, was observed in young mice compared to older mice. Restoring this ratio in older mice through FMT improved outcomes following a stroke [66].

While the research on the gut microbiota and brain axis in stroke is still in its early stages, the studies suggest that the gut microbiome may be a promising target for stroke prevention and treatment. However, more research is needed to fully understand the mechanisms by which the gut microbiota impacts stroke and to develop effective strategies for manipulating the gut microbiota to prevent and treat stroke patients.

## 6. High Altitude

Acute mountain sickness, high-altitude cerebral edema, and high-altitude pulmonary edema are some of the well-documented complications of rapid ascent to high altitude. Cerebrovascular diseases have also been reported at high-altitude, mostly in the form of small descriptive studies, though there are more published data related to cerebral venous thrombosis (CVT) as compared to stroke [67,68,69,70]. Altitudes are categorized into six levels: low (less than 1500 m), intermediate (1500–2500 m), high (2500–3500 m), very high (3500–5800 m), extreme high (above 5800 m), and the “death zone” (above 8000 m) [71].

Studies reporting the impact of higher altitudes on stroke have yielded inconsistent findings. One of the reasons for inconsistency is the limited stroke epidemiological data of the long-term high-altitude inhabitants, even though >5% of the worldwide population lives above 1500 m and millions of people are chronically exposed to hypobaric hypoxia. Cerebral autoregulation is compromised at high altitudes, affecting not only individuals residing chronically at high altitudes but also newcomers to such environments [72]. High altitudes also lead to various physiological adaptations in the human body, including, but not limited to, elevated hemoglobin and hematocrit levels, lung volume changes, and heightened orthostatic tolerance [73].

Several studies have reported an increased risk of thrombosis and ischemic stroke at high altitudes, mostly above 2500 m [74,75,76,77,78,79]. These patients living at high altitudes with ischemic stroke were predominantly male and were of younger age, with a mean age of 40 years at the time of stroke diagnosis [74,75,77,78,79,80]. A study by Lu et al. in 2020, comparing patients with acute ischemic stroke (AIS) in Tibet (higher altitude of 3650 m) and Beijing (lower altitude of 40 m), found that the patients diagnosed with AIS in Tibet were generally younger than those in Beijing (58.19 vs. 65.10 years; *p* < 0.001) [79]. One study specifically looking at the radiologically proven strokes found that among 4000 high-altitude residents under 40 years, there were 10 radiologically proven strokes, compared to only 1 among 4000 low-altitude residents of the same age group [74]. There are multiple plausible explanations for these effects. A decrease in atmospheric pressure at high altitudes causes hypoxia that may result in compensatory hyperventilation, tachycardia, vasoconstriction, erythrocytosis, and increased blood viscosity increasing the risk for thromboembolic complications [79].

On the other hand, a Swiss ecological study in 2009 reported decreased stroke mortality at higher altitudes (259–1960 m), with a 12% decreased stroke mortality per 1000 m gained in elevation. These results were statistically significant even after adjusting for potential vascular confounders [81]. Additionally, three separate ecological studies from the United States, Austria, and Ecuador, respectively, also reported similar results that living at moderately high altitudes (<3500 m) have a beneficial effect on ischemic heart disease and stroke mortality [82,83,84]. The protective effects were more pronounced within the altitude range of 2000 to 3500 m [84]. An observational study in sub-himalayan region of India also reported a reduction in stroke incidence among hospitalized patients at a moderate altitude of 2000 m [85]. The possible explanations for these effects could be a better adaptation to hypoxia and the involvement of hypoxia-inducible factor (HIF), which is a transcription factor and has been found to provide resistance against ischemia and improve cardiac function. HIF senses cellular hypoxia and helps cells adapt and survive in a hypoxic environment by upregulating erythropoietin, promoting angiogenesis, and increasing glycolysis [86].

Air travel is now a common occurrence, with nearly 2 billion people traveling annually [87]. Fortunately, flight-related strokes are rare. Commercial planes typically fly at an average peak altitude ranging from 33,000 to 42,000 feet (~10,000 to 12,800 m), while still maintaining the intra-cabin air pressure [88]. However, flights have been associated with an increased risk of deep venous thrombosis and pulmonary embolism, a connection often referred to as the “economy class syndrome” [87,89]. This is thought to be due to paradoxical embolism through a patent foramen ovale (PFO) [89], although the role of PFO in ischemic stroke even in the general population remains unclear. Humaidan et al. published a case series of 42 patients with flight-related strokes and found paradoxical embolism in only 17% of cases, a figure lower than expected. They observed a wide range of etiologies similar to those seen in general stroke patients [87]. Another case series of 44 patients (32 with AIS, 12 with Transient Ischemic Attacks) revealed paradoxical emboli in 18% of their patients. They also noted a higher frequency of high-grade carotid stenosis and occlusion in their cohort (12 patients, 27%). Furthermore, 67% of the strokes linked to high-grade carotid stenosis or occlusion occurred on longer flights (>4 h) [90]. It is hypothesized that in individuals with already compromised carotid perfusion, the combination of dehydration and a decrease in cabin air pressure leading to hypoxemia may increase the risk of stroke [90].

Our present knowledge about altitude and stroke indicates that high altitudes of >3500 m increase the risk of ischemic stroke but when people reside between 1500 to 2500 m, there appears to be a protective effect for stroke, possibly due to better adaptation to hypoxia [82,83,84,91]. However, it is unclear at what point this protective effect turns into a risk factor. It is also important to note that the observed differences between residents of high and low altitudes could potentially be influenced by genetic variations. In conclusion, the association between high altitude and stroke is complex and elusive. We need large-scale studies to firmly establish the connection between altitude and stroke.

## 7. Systemic Infections

There is growing evidence that supports a link between infections, acute or chronic, and the development of atherosclerosis and stroke [92,93,94,95,96,97,98,99,100]. Various infections have been associated with stroke, including but not limited to infective endocarditis [93], tuberculous meningitis (TBM) [92], Chagas disease [94], HIV infection [95], varicella zoster infection [95], chronic hepatitis infection [96,97], and most recently, coronavirus disease 2019 (COVID-19) [98].

According to a hospital database in New York, all types of infections increase the risk of AIS, with urinary tract infections posing the highest risk for both AIS (OR 5.32) and hemorrhagic stroke (OR 1.80). Additionally, recent respiratory infections increase the risk of subarachnoid hemorrhage (OR 3.67) [99]. Another study reported that more than 8% of inpatient stroke patients had sepsis preceding stroke, with ORs of 28.36 and 12.10 for AIS and hemorrhagic stroke, respectively, within a 15-day period of sepsis diagnosis [100].

Infective endocarditis is a well-known risk factor for cerebrovascular diseases, predominantly manifesting as ischemic stroke [93,101,102,103]. Gram-positive organisms, particularly Staphylococcus aureus and Streptococcus bovis, are the predominant pathogens isolated within this population [103]. Septic emboli, during their transit to the cerebrum, may result in small, scattered infarctions across multiple vascular territories or, in some instances, obstruct a major vessel such as the anterior or middle cerebral artery. These infarctions are also highly susceptible to hemorrhagic conversion due to increased vascular friability [101,102]. Patients with infective endocarditis and stroke have been reported to have an increased acute inpatient mortality rate (15–20%), with up to 40% experiencing mortality within the first 5 years post-stroke [101,102,103]. Additionally, cerebral microbleeds are frequently observed in infective endocarditis patients (up to 57%), in contrast to the general population (~5%). Cerebral microbleeds are mostly cortical in location and they represent vascular frailty. Cerebral microbleeds were also reported to be a strong predictor for future hemorrhagic stroke in these patients [104]. 

Acute bacterial meningitis and viral encephalitis are also not uncommon to precipitate stroke as their complication [102]. The proposed pathophysiology often involves vasculopathy, either in the form of vasculitis or vasospasm, along with intravascular coagulation-induced thrombosis. Similar to bacteria, viruses have also been implicated in the etiology of stroke. Notably, HIV [95], herpes viruses, hepatitis viruses [96,97], and more recently, SARS-CoV-2 [98], are frequently identified in this context. Within the herpes virus family, herpes simplex virus types 1 and 2, as well as Varicella Zoster virus (VZV), are commonly linked to strokes in both pediatric and adult populations [105]. The diagnosis of VZV-associated stroke can pose challenges, especially if a rash does not precede the onset of the stroke [105]. Among hepatitis viruses, chronic hepatitis B and C infections have also been associated with strokes, notably chronic HCV infection [97]. The incidence of stroke in HCV-infected patients has been reported to be approximately 25% higher than controls. In these patients, the presence of mixed cryoglobulinemia is one of the main factors precipitating ischemic stroke [97]. A large Korean study (*n* = 521,421) reported the association between stroke and chronic hepatitis B infection, but the association is linked to liver dysfunction rather than HBsAg seropositivity alone [96]. 

Apart from common viral and bacterial infections, TBM is a chronic infection that is prevalent in developing countries and associated with cerebral infarctions [92]. A retrospective study consisting of 559 TBM patients reported acute and subacute brain infarctions in one-quarter (25.8%) of patients based on brain imaging. The presence of infarctions was identified as an independent predictor of mortality. The study also reported that the risk of cerebral infarctions increased in patients aged above 40 years (adjusted OR (AOR), 1.7) and in the presence of vascular comorbid conditions such as dyslipidemia (AOR, 9.7), diabetes mellitus (AOR, 2.1), and hypertension (AOR, 1.8) in this population [92]. TBM-associated strokes are usually subcortical and have a predilection for certain brain areas that include basal ganglia, internal capsule, and thalamus [106]. Basal inflammatory exudates, vasculitis, vasculospasm, initial proliferation, and prothrombotic state are some of the proposed mechanisms [92].

Fungal infections are uncommon causes of stroke, but they can have high mortality rates in immunocompromised individuals [107,108]. These patients usually present with recurrent or progressive strokes and are erroneously labeled with cryptogenic strokes before confirming the fungal infection [107,108,109]. Aspergillus and Mucormycetes may lead to stroke through vasculitis, mycotic aneurysms, vascular necrosis, and direct invasion, while Cryptococcus and Candida cause small vessel vasculitis and subpial ischemic lesions [108]. Certain fungi have predilection for vascular territories, for instance, Aspergillus usually affects the anterior circulation, and Dematiaceous fungi like Exserohilum rostratum, which is linked to contaminated glucocorticoid injections for chronic pain, usually affects the posterior/vertebro-basilar circulation [107,109].

Chagas disease, a parasitic infection common in South America, frequently causes cardiomyopathy and is also a risk factor for ischemic stroke, mostly due to the cardioembolic phenomenon [94]. A retrospective autopsy study has reported that up to one-third (10–35%) of patients with Chagas disease have cerebral infarctions [110]. Another autopsy study reported that not only cerebral infarctions were common in these patients, but they were also associated with mortality in more than half (52%) of these patients [111]. However, limited data are available to project the cumulative risk of ischemic stroke in patients living with Chagas disease.

Recently, COVID-19 infection has made headlines due to its reported association with increased AIS cases, as well as poor functional outcomes. A comparative study of AIS patients, stratified by COVID-19 status, revealed that individuals with a positive COVID-19 status were at a substantially higher risk of experiencing a poor functional outcome at discharge, defined as a modified Rankin scale (mRs) score of 3 or more. The observed association persisted even after accounting for various confounding factors, resulting in a 3.87-fold increase in the odds for the adverse outcome. Furthermore, COVID-19 infection emerged as a significant independent predictor of inpatient mortality [98].

The pathophysiology of infections leading to stroke is multifaceted, with key factors including accelerated atherosclerosis, changes in pro- and anti-thrombotic factors, thrombocytopenia, platelet dysfunction, the production of autoantibodies, direct vessel wall damage, or indirect damage mediated by an inflammatory response leading to vasculitis, aneurysm, and thromboembolism [92,95,96,97,112,113]. The inflammatory response is triggered by cytokines and acute phase proteins like heat shock proteins [101,102]. Chronic infections not only independently increase the risk of developing carotid artery atherosclerosis, even in individuals without preexisting atherosclerosis at baseline (OR, 4.08) but also exacerbate atherogenesis in high-risk patients with underlying vascular comorbidities [113]. 

## 8. Conclusions

This article summarizes emerging non-traditional risk factors associated with stroke, including both ischemic and hemorrhagic strokes. All these risk factors are modifiable. We need to make a conscious effort addressing them with our patients and creating awareness at a larger scale on a public level.

## Data Availability

No new data were created or analyzed in this study.
